# High-dimensional single-cell analysis delineates radiofrequency ablation induced immune microenvironmental remodeling in pancreatic cancer

**DOI:** 10.1038/s41419-020-02787-1

**Published:** 2020-07-27

**Authors:** Qinglin Fei, Yu Pan, Wenji Lin, Yuanyuan Zhou, Xingxing Yu, Zelin Hou, Xunbin Yu, Xianchao Lin, Ronggui Lin, Fengchun Lu, Hongdan Guan, Heguang Huang

**Affiliations:** 1 Department of General Surgery, Fujian Medical University Union Hospital, Fuzhou, 350001 China; 2 Department of Radiology, Quanzhou First Hospital of Fujian Medical University, Quanzhou, 362000 China; 3 Department of Geriatrics, Fujian Medical University Union Hospital, Fuzhou, 350001 China; 4 Department of Pathology, Fujian Provincial Hospital, Fuzhou, 350001 China; 5 Department of Radiation Oncology, Fujian Medical University Union Hospital, Fuzhou, 350001 China

**Keywords:** Cancer microenvironment, Tumour immunology

## Abstract

Radiofrequency ablation (RFA) is an effective local therapy approach for treating solitary tumor of many types of malignancy. The impact of RFA on the tumor immune microenvironment on distant tumors after RFA treatment is still unclear. In this study, by using syngeneic tumor models and single-cell RNA and T-cell receptor sequencing, we have investigated the alterations of tumor-infiltrating immune cells in distant non-RFA tumors. Single-cell RNA sequencing identified six distinct lymphoid clusters, five distinct monocyte/macrophage clusters, three dendritic cells clusters, and one cluster of neutrophils. We found that RFA treatment reduced the proportions of immunosuppressive cells including regulatory T cells, tumor-associated macrophages and tumor-associated neutrophils, whereas increased the percentages of functional T cells in distant non-RFA tumors. Moreover, RFA treatment also altered gene expressions in single-cell level in each cell cluster. By using pseudo-time analysis, we have described the biological processes of tumor-infiltrating CD8^+^ T cells and monocytes/macrophages based on the transcriptional profiles. In addition, the immune checkpoints including PD-1 and LAG3 were upregulated in the T cells in distant non-RFA tumors after RFA treatment. In conclusion, our data indicate that RFA treatment induced remodeling of tumor immune microenvironment in distant non-RFA tumors in pancreatic cancer mouse model and suggest that combining RFA with immune checkpoint inhibitors may be an effective treatment approach.

## Introduction

Pancreatic ductal adenocarcinoma (PDAC) is the fourth leading cause of cancer-related mortality in the United States^1^. Most patients diagnosed with an advanced stage with limited effective treatment options^2^. Immune checkpoint inhibitors have had remarkable efficacy in many malignancies, such as advanced melanoma, non‐small‐cell lung cancer, renal cell carcinoma, bladder cancer, Hodgkin lymphoma, and head and neck cancer^3,4^, but not in PDAC. The immunosuppressive tumor microenvironment (TME) in PDAC, characterized by typically poor infiltration of effector T cells and prominent myeloid inflammation, appears to be the main factor for such treatment resistance^5^. Studies from our laboratory^6^ and other laboratories^4,7–10^ have revealed that the combination of immune checkpoint inhibitors and other modalities, including radiotherapy, chemotherapy, oncolytic virus, targeted therapy, and other immunotherapies, may be a promising strategy.

Radiofrequency ablation (RFA) is a local therapy effective for oligometastasis of lung and liver as well as other organs^10–12^, RFA induced localized coagulative necrosis that may lead to the release of tumor antigens, which could elicit systemic adaptive immune response against the tumors^10^. It is not clear if the immune response induced by local RFA could generate effective antitumor immune responses in distant non-RFA tumors^13^. Most studies have focused on the changes of immune microenvironment in the RFA-treated tumors after RFA treatment^14^.

**Fig. 1:**
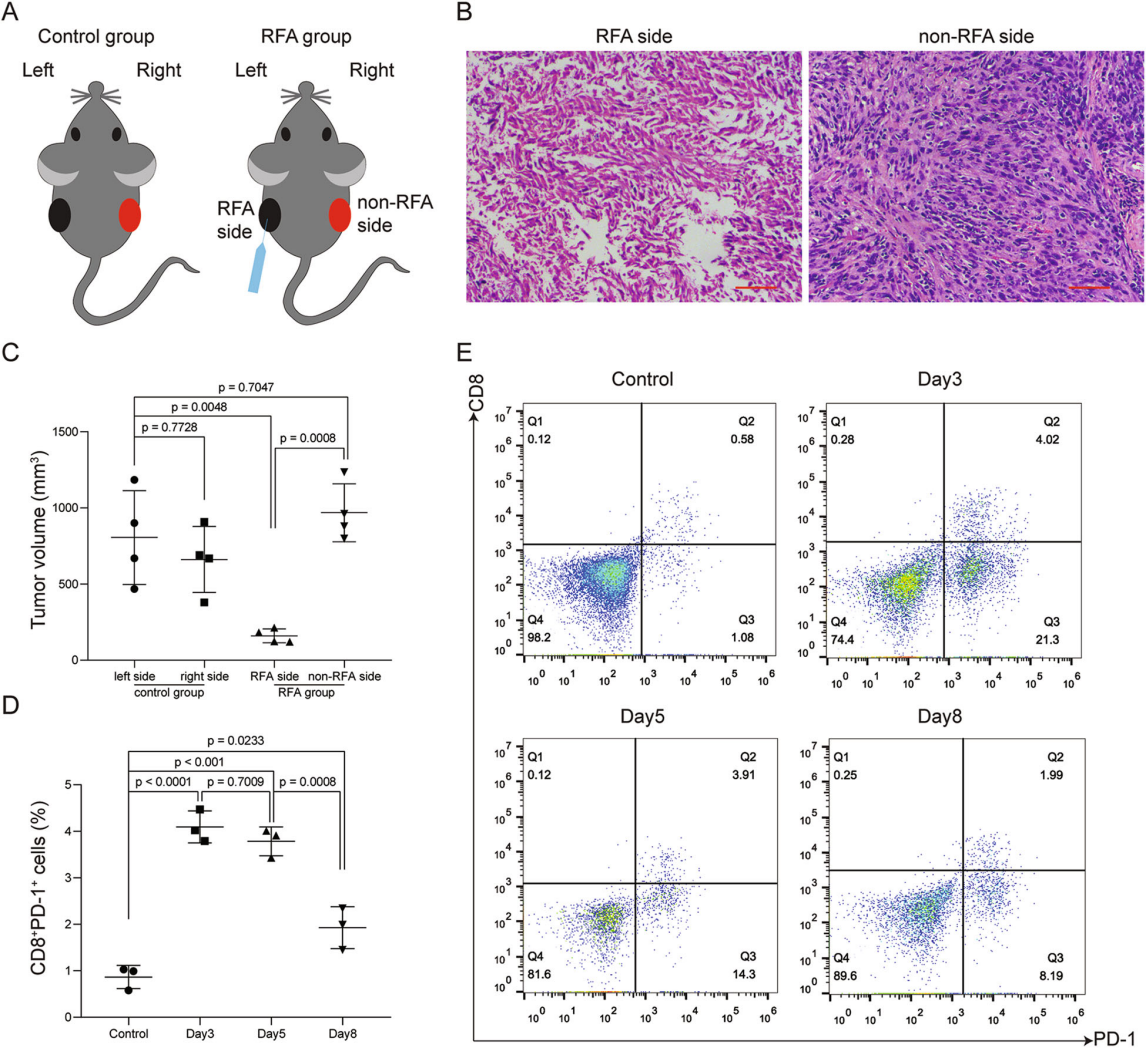
Changes in distant non-RFA tumor after RFA treatment. **a** Diagram of control group and RFA group. RFA treatment was performed on the left side tumors (RFA side). The right side (non-RFA side) tumors were used to further analysis. **b** H&E staining of tumor tissues on RFA and non-RFA side after RFA treatment. Scale bar = 50 μm (red line at the bottom right). **c** After 2 weeks, tumors from control group and RFA group were harvested and measured. Tumor volumes were compared to each two groups. *p* values were calculated based on a Student’s *t* test (*n* = 4 per group). **d**, **e** Percentage of CD8^+^PD-1^+^ cells in distant non-RFA tumor on day 3, 5, and 8 after RFA treatment. *p* values were calculated based on a Student’s *t* test (*n* = 3 per group).

We have established a PDAC mouse model with tumor implant on bilateral flanks to recapitulate RFA and non-RFA sides (Fig. 1a). After RFA treatment on one side of tumor, the single-cell RNA sequencing (scRNA-seq) and single-cell T-cell receptor (TCR) sequencing were used to evaluate the cellular compositions and transcriptional landscape of TME on the other side, the non-RFA tumor. We have used the single-cell transcriptomics to characterize the changes of immune cells in response to RFA in the TME.

## Materials and methods

### Tumor transplantation and treatment

The mouse pancreatic cancer cell line Panc02 was obtained from Shanghai Aolu Biological Technology Company. Tumor cell propagated in vitro in Dulbecco’s Modified Eagle Medium (Hyclone) supplemented with 10% fetal bovine serum (FBS, Gibco) were washed extensively, resuspended in endotoxin-free phosphate-buffered saline (PBS) and then 200 µl injected (1 × 10^6^ cells) subcutaneously into male C57BL/6 mice on bilateral flanks, respectively. Then randomly separated into two groups and no blinding was done. In this study, 3–5 mice per group were used as indicated in the Figure legend. Treatments were initiated when the tumor volume reached ~500 mm^3^.

RFA was performed using an 18-gauge single ablation electrode (STARmed Co., Ltd., Korea) with 7 mm active tip inserted percutaneously and orthogonal to the skin in the center of the tumor on the left flank, whereas the tumor on the right side did not receive any intervention. Treatments were administered for 3.5–4.5 min at the target temperature of 70 °C to ensure complete ablation of the target tumors^13^. The tumor on the right flank (non-RFA side) was used for further analysis (Fig. 1a). All experiments were approved by the Ethics Committee for Animal Research of 900 Hospital of the Joint Logistics Team.

### Tumor harvest

Tumor masses on the right flank were removed, diced, digested with tumor dissociation kit (Order no. 130–095–929, Miltenyi Biotec Inc.) at 37 °C for ~40 min. Resuspend sample and applied the cell suspension to a 70 μm MACS SmartStrainer (Order no. 130–098–462) to remove debris and separate cell clumping. Cells were washed twice. Red blood cells were lysed using ACK lysis buffer (GS3309). To remove aggregates and clumps, cells were filtered through a 70 μm MACS SmartStrainer. Then cells were washed and resuspended in PBS plus 1% FBS for analysis.

### Flow cytometric analysis

Antibodies against CD8a (53–6.7, eBioscience), PD-1 (29 F.1A12, BioLegend) were used for marker staining for 30 min at 4 °C. All flow cytometry was performed on the BD Accuri C6 Plus (BD Biosciences) and analyzed using FlowJo software.

### Immunohistochemistry

Immunohistochemical experiment procedure was described previously^15^. The rabbit anti-CD4 antibody (EPR19514, Abcam), anti-CD8 antibody (YTS169.4, Abcam), rabbit anti-FOXP3 antibody (D2W8E, Cell Signaling Technology), rabbit Anti-CD206 antibody (ab64693, Abcam), and rabbit anti-iNOS antibody (ab15323, Abcam) were used to immunohistochemistry staining. The density of the positive cells was calculated with ×400 magnification in five representative fields in the tumor tissues, and the average was calculated. Each section was examined independently by two investigators in a blinded manner.

### Intratumoral CD45^+^ cell isolation

For separating of intratumoral CD45^+^ cells, single-cell suspensions of digested tumor were resuspended in a buffer (PBS, 0.5% bovine serum albumin, and 2 mm thylenediaminetetraacetic acid) and labeled with CD45 (TIL) MicroBeads (Order no. 130‐110‐618, Miltenyi Biotec Inc.) for 15 min in the dark at 4 °C. CD45^+^ cells were separated whit LS columns (order no. 130‐042–401, Miltenyi Biotec Inc.). The purity of the separated cells was used for scRNA-seq analysis.

### Single-cell RNA-seq

Single-cell RNA-seq library preparation: according to the manufacturer’s instructions, single-cell RNA-seq was performed by encapsulating separated live CD45^+^ tumor-infiltrating cells into droplets and libraries were constructed using Single Cell 5′ Library and Gel Bead Kit and Single Cell V(d)J Enrichment Kit. The libraries were finally sequenced using an Illumina Novaseq6000 (performed by CapitalBio, Beijing).

### Cell ranger pipeline

The Cell Ranger software (3.0.1) obtained from 10× Genomics website https://support.10xgenomics.com/single-cell-gene-expression/software/downloads/latest was used to perform sample demultiplexing, alignment, filtering, barcode counting, and UMI counting. Cellranger mkfastq was used to demultiplex raw base call files into sample-specific fastq files. Then, fastq files for each sample were processed with Cellranger count, which was used to align samples to mm10 genome, filter and qualify. For each sample, the recovered-cells parameter was specified as 8000 cells that we expected to recover for each individual library. The data for each sample were aggregated using the cellranger aggr pipeline, which performed a between-sample normalization step and merged two samples into one.

### Seurat package analysis

The Seurat pipeline^16,17^ was applied to the analysis of the combined data set. Genes that were expressed in less than three cells and cells that expressed <400 and >5500 genes, were excluded. The function Sctransform was used for normalization. Principle component analysis (PCA) was performed on ~3000 genes with PCA function. A UMAP dimensional reduction was performed on the scaled matrix (with most variable genes only) using first 40 PCA components to obtain a two-dimensional representation of the cell states. Cell clustering was performed using the function FindClusters that implements SNN (shared nearest neighbor) modularity optimization base clustering algorithm on 40 PCA components with resolution 0.8, leading to 17 clusters.

### Identification of cluster-specific genes and marker-based classification

To identify marker genes, the FindAllMarkers function was used with the MAST test for single-cell gene expression. For each cluster, only genes that were expressed in >25% of cells with at least 0.25-fold difference were considered^18^. To characterize clusters, we used the ImmGen. To perform pathway analysis, we used the R package ClusterProfiler^19^ to compared clusters inside cohorts (e.g., T cells, macrophages) with different parameters (0 fold and threshold of at least 10% of cells, expressing this gene inside cluster) to find the differential expression markers. For heatmap representation, mean expression of markers inside each cluster was used.

### Lymphoid population analysis

To further explore lymphoid cells, clusters expressing *Cd3d* were extracted from aggregated samples. Most variable genes, PCA, UMAP, clustering (resolution 1 on 40 first PCAs) and marker selection analysis was performed as described above.

### Statistical analysis

At least three biological replicates were used in each experiment unless otherwise stated. Two tail Student’s *t* tests and one-way ANOVA were used for analyzing the quantitative data. A *p* < 0.05 was statistically significant. All statistical analyses were performed by SPSS software version 22.

## Result

### RFA induced transient immune responses in distant non-RFA tumor in pancreatic cancer mouse models

To assess the efficacy of RFA in pancreatic cancer, syngeneic Panc02 mouse models were established and divided into two groups: treatment with or without RFA (RFA group and control group, Fig. 1a). The methods of RFA treatment were described in the “Methods” section. All tumors were harvested and measured after 2 weeks. We found that the tumor growth of RFA side in RFA group was significantly reduced compared with the control group, whereas the tumor growth of non-RFA side in RFA group did not change significantly (Fig. 1c). Hematoxylin and eosin staining indicated that the areas of coagulative necrosis in tumor tissues of RFA side in RFA group (Fig. 1b). To understand the duration of immune response in the tumors of non-RFA side in RFA group, we measured the tumor-infiltrating CD8^+^PD-1^+^ T cells of non-RFA side on days 3, 5, and 8 after RFA treatment by flow cytometry. We found that the percentage of CD8^+^PD-1^+^ T cells increased compared with the control group on day 3 (Fig. 1d, e), began to decrease slightly on day 5 and showed marked decrease on day 8 (Fig. 1d). These results demonstrated that in the early stage (3–5 days) after RFA treatment, the RFA-induced immune responses were observed in the non-RFA distant tumors, but transient and failed to suppress the tumors.

### Identification of tumor-infiltrating immune cells by using scRNA-seq

**Fig. 2:**
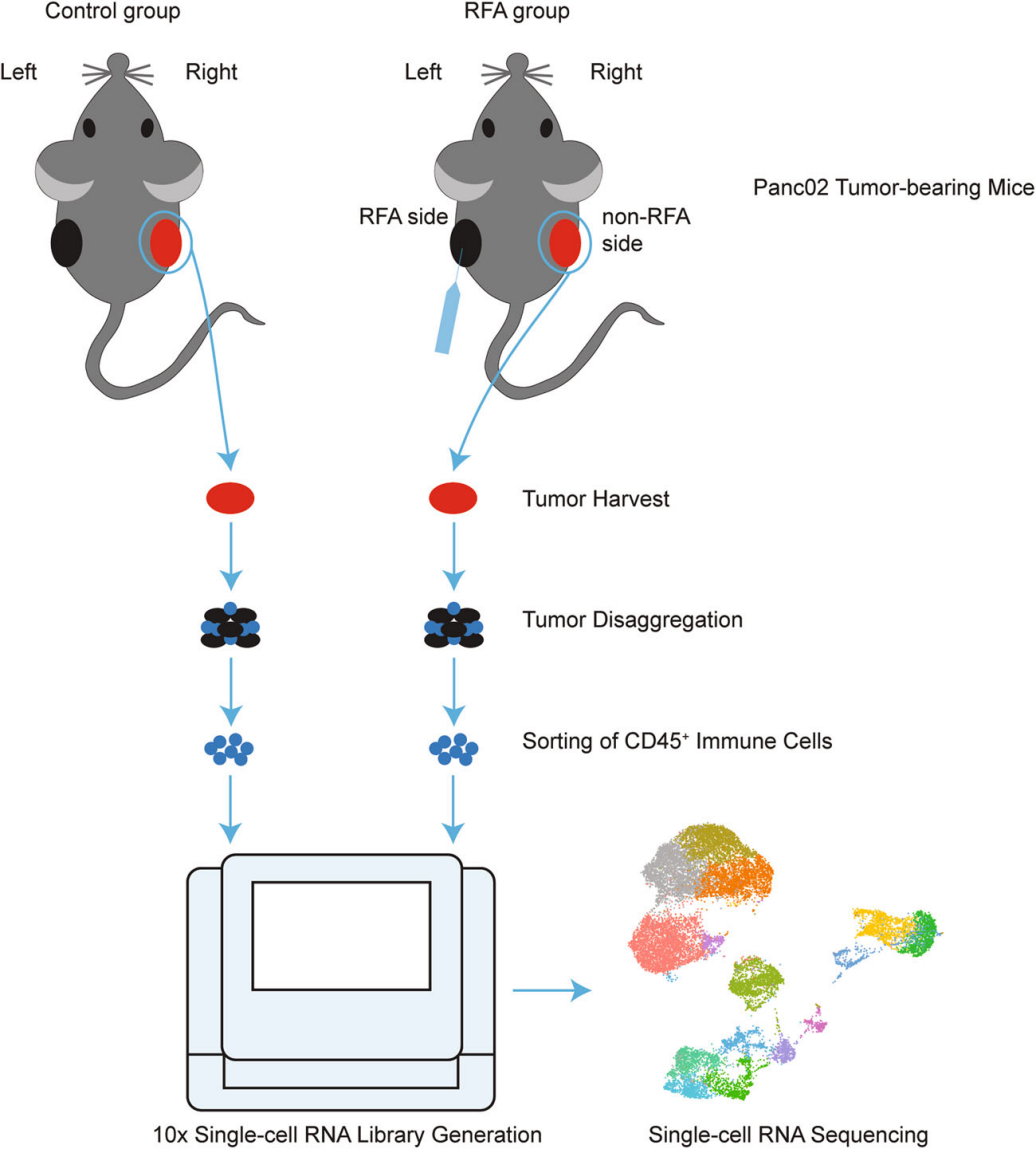
Experimental setup. Syngeneic Panc02 mouse models were established and divided into two groups. RFA Treatments were initiated on the left side in RFA group when the tumor volume reached ~500 mm^3^, whereas the tumor on the right side did not receive any intervention. Tumors were harvested on day 3 after RFA treatment, and then digested and sorted for CD45^+^ immune cells. CD45^+^ immune cells were pooled together from three mice in each group and then subjected to scRNA-seq and TCR sequencing using a 10× Genomics pipeline.

To further characterize the RFA-induced changes on the tumor-infiltrating immune cells of the non-RFA distant tumors, we used high-dimensional scRNA-seq analysis to reveal the complexity of tumor immunity. We obtained the CD45^+^ immune cells from tumor mass and analyzed them by scRNA-seq and single-cell TCR sequencing with a 10× genomics pipeline (Fig. 2).

**Fig. 3:**
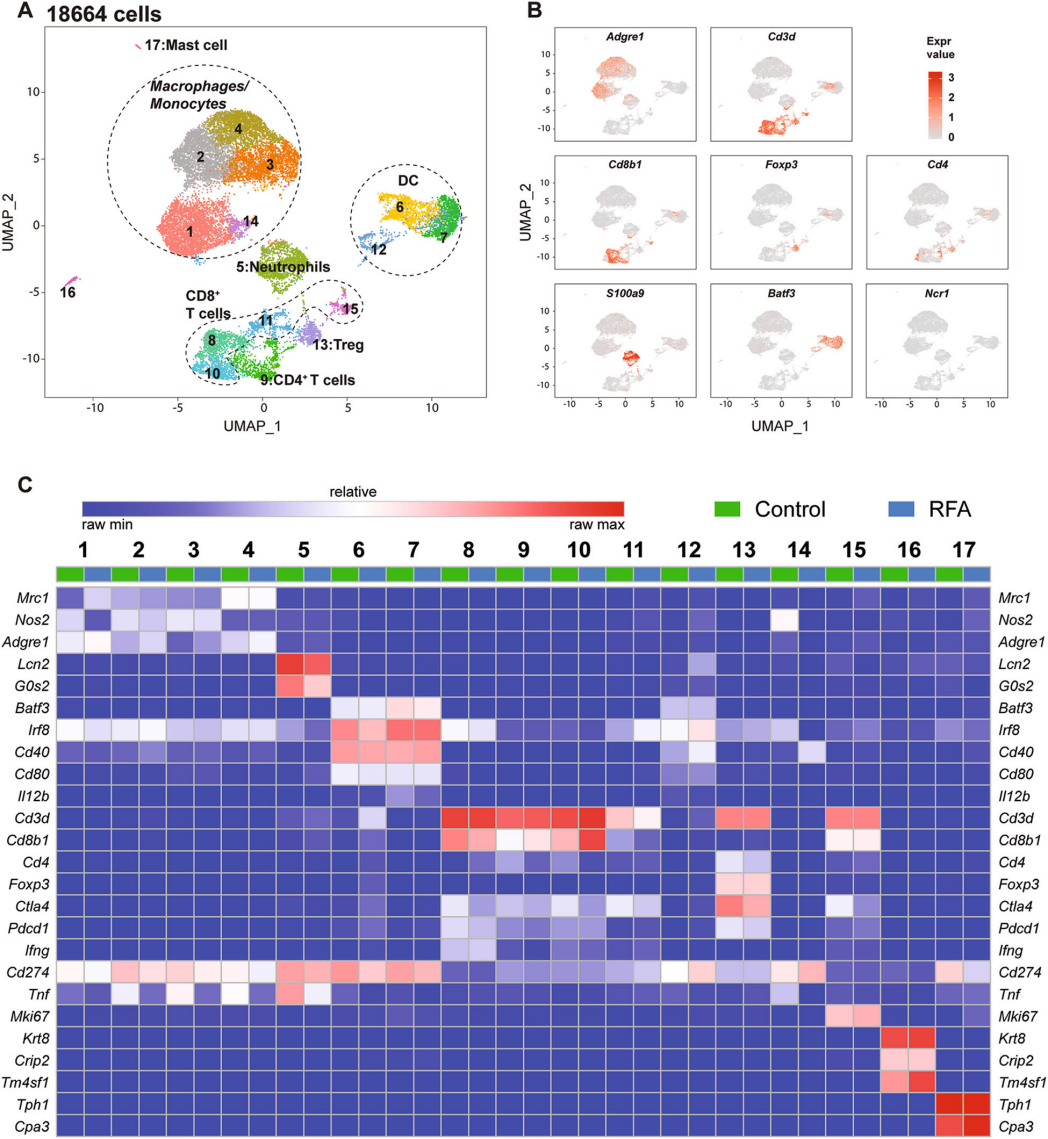
Identification of cell populations based on single-cell gene expression in intratumoral CD45^+^ immune cells. **a** UMAP plot of cells from two groups aggregated. **b** UMAP plot of immune cells displaying select marker-gene expression. **c** Heatmap displaying normalized expression for selecting genes in each cluster.

We performed scRNA-seq analysis on CD45^+^ cells isolated from the tumor tissues of control group (*n* = 10,659 cells sequenced, coverage of 84 655 mean reads per cell) and the non-RFA side of the RFA-treated group (*n* = 8005 cells sequenced, coverage of 112,967 mean reads per cell). All cells (*n* = 18,664) were clustered into unbiased cell type classifications (Fig. 3a) by using the Seurat single-cell analysis R package^16,17^. Comparison with the ImmGen database and assessment of known cell type markers identified six distinct lymphoid clusters, five distinct monocyte/macrophage clusters, three clusters of dendritic cells (DCs), and one cluster of neutrophils (Fig. 3a–c). Clusters are annotated as “XXX_s#” where “XXX” represents the cell types, “s” represents the scRNA-seq, and “#” the different clusters.

### Use of scRNA-seq and single-cell TCR sequencing to identify RFA-induced changes in the tumor-infiltrating lymphocytes

**Fig. 4:**
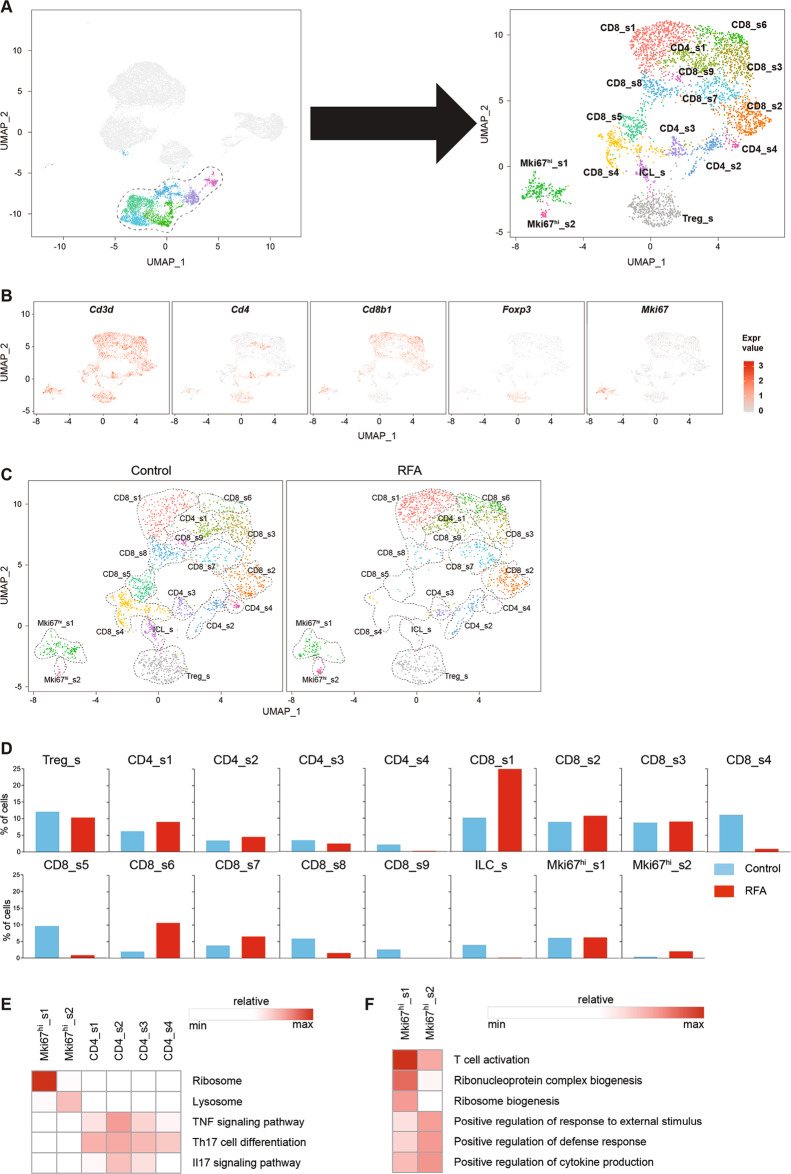
RFA induces remodeling of tumor-infiltrating lymphoid cells. **a** UMAP plot showing the merged data of intratumoral lymphocytes. **b** UMAP plot of lymphoid cells displaying select marker-gene expression. **c** Comparison of each cluster in two groups. **d** Percentage of cells in each cluster by condition. **e** Heatmap of gene ontology identifying pathway enrichment by each subpopulation. **f** Heatmap of KEGG enrichment analysis enrichment by clusters.

To elucidate the transcriptional and functional changes in lymphoid cells, we isolated the lymphoid cells from the tumors by separating them from the other immune cells and reanalyzed them at a high resolution (Fig. 4a). This strategy yielded 17 distinct lymphoid subpopulations broadly defined by the distribution of the classical marker genes (Fig. 4b). We then performed an integrative analysis of scRNA-seq and TCR repertoire in the lymphoid subpopulations to examine the cellular context of the immune responses and immune repertoires of T cells. The clonotypes were defined based on the CDR3 sequences of both TCR alpha and beta chains using the cell ranger V(D)J analysis pipeline^20^. We eliminated the non-productive and low-abundance TCRs. Subsequently for defining the clonotype, we strictly defined T cells with at least one pair of identical paired alpha and beta chains to be one clonotype. In total, we detected 641 clonotypes among 1236 cells in control group and 605 clonotypes among 1099 cells in RFA group. The details of clonotypes in each cluster of lymphoid subpopulations were shown in Table S1. Using this analysis approach, RFA-induced changes in the subpopulation proportions and their transcriptional profiles could be exhibited clearly in each cell type.

### Dysfunction of Regulatory T cells (Tregs)

**Fig. 5:**
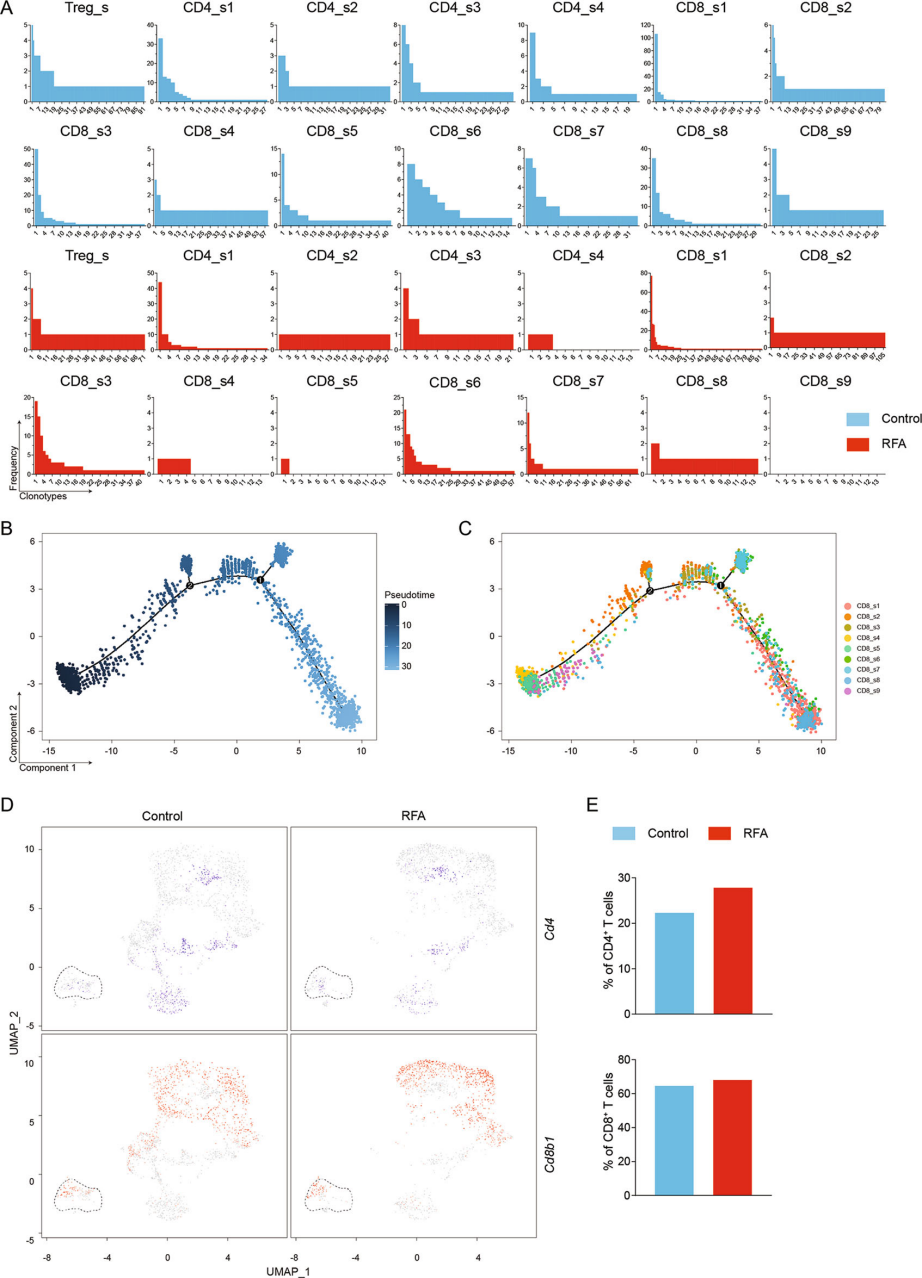
Quantitative and qualitative changes of lymphocytes in individual clusters. **a** Occupancy of clonotypes in each cluster by condition. **b** Pseudo-time trajectory of CD8^+^ T cells analyzed using Monocle 2. **c** CD8^+^ T-cell clusters from scRNA-seq overlaid on Monocle 2 pseudo-time plot. **d** UMAP plots showing CD4 (blue) and CD8 (red) expression in Mki67^hi^_s1 by condition. **e** Percentage of CD4^+^ and CD8^+^ T cells in Mki67^hi^_s1 by condition.

Tregs express *Foxp3* and *Cd4*, and play immunosuppressive roles in the TME^21^. We found that the number and percentage of Tregs were marginally decreased in RFA group compared with the control group (Fig. 4d). Moreover, by using immunohistochemical staining, we revealed that the number of FOXP3-positive cells in the tumor tissues were similar between the RFA and the control group (Figure S2). After RFA treatment, Tregs displayed increasing expression of *Foxp3* and *Il2ra* (Figure S1), which may stabilize and sustain Tregs by signaling through the IL-2/IL-2R axis^22^, suggesting Tregs’ immune suppression was enhanced. However, expressions of *Il6ra*, *Tnfrsf4*, *Tnfrsf18*, and *Ccl3* (Figure S1) were also increased in the RFA group. The activation of OX40 (*Tnfrsf4*), glucocorticoid-induced TNF receptor-related gene (*Tnfrsf18*) and IL-6/IL-6R axis may adversely affect the stability of the Treg phenotype^22^. In addition, Tregs also require activation via the TCR for full acquisition of their immunosuppressive function^23^. The TCR analysis indicated that the multiformity and quantity of clonotypes were reduced after RFA treatment (Fig. 5a). In sum, the RFA-induced transcriptional and functional changes may reduce the immunosuppressive function of Tregs.

### Changes in CD4^+^ T cells

Single-cell RNA-seq identified four distinct clusters of FOXP3^-^CD4^+^ T cells (CD4_s1, CD4_s2, CD4_s3, CD4_s4) (Fig. 4c). Both CD4_s3 and CD4_s4 expressed T-cell activation markers *Cd81*, *Stat3*, *Rora*, and *Tgfb1* (Figure S1), however, these two clusters also expressed higher levels of genes associated with the exhausted T cells, such as *Pdcd1*, *Ctla4*, and *Icos*^18^. CD4_s2 had higher expression of *Traf1*, *Ifngr1*, *Il2ra*, *Il17a*, *Il17f*, and *Cxcl3* (Figure S1). These genes are part of the TNF-signaling pathway, Th17 cell differentiation, and Il17 signaling pathway (Fig. 4e). CD4_s1 exhibited high expression of cytotoxic molecules, such as *Nkg7* and *Gzmb*, and also expressed high level of *Ifng*, *Eomes*, and *Prf1* (perforin), suggesting the immune cells in CD4_s1 may be cytotoxic CD4 T cells^20,24^. In addition, RFA treatment decreased the number of CD4_s3 and CD4_s4 cells and increased the number of CD4_s1 and CD4_s2 cells (Fig. 4d). Using immunohistochemical staining, we also observed that the number of CD4^+^ T cells increased after RFA treatment (Figure S2). The available TCR sequences for these cells revealed that CD4^+^ T-cell clusters had a similar number of clonotypes between control group and the RFA group (Fig. 5a). Based on the different numbers of cells in each clonotype of each cluster, we calculated the percentage and the number of cells in clonotype 1–5 (Table S2). We found that compared with the other clusters, CD4_s1 occupied clonotype 1–5 were highest in both control and RFA group (Fig. 5a and Table S2). These findings demonstrate the augmentation of CD4^+^ T-cell activation, especially cytotoxic CD4 T cells, was triggered by RFA treatment.

### Transformation in CD8^+^ T cells

CD8^+^ T cells are also known as cytotoxic T cells, which induce apoptosis of target cells by releasing the cytotoxins perforin, granzymes, and granulysin or Fas–Fas ligand signal molecules. In this study, scRNA-seq revealed nine distinct subsets of CD8^+^ T cells (Fig. 4c) and majorities of them were cytotoxic T cells. T cells in the CD8_s1, CD8_s8, and CD8_s9 clusters expressed higher level of several functional markers, like *Gzmb*, *Prf1*, and *Fasl* (Figure S1). CD8_s3, CD8_s6, and CD8_s7 clusters had high expression of *Gzmb* and median expression of *Fasl*. Almost all these cytotoxic T cells expressed exhaustion genes [*Havcr2* (*Tim3*), *Lag3*, *Pdcd1*, *Ctla4*]. The other three clusters (CD8_s2, CD8_s4, CD8_s5) expressed naive T-cell markers *Sell* (CD62L) and *Ccr7*^25^, however, these cells also expressed median levels of *Fasl*. Following RFA treatment, the proportions of CD8_s1, CD8_s2, CD8_s6, and CD8_s7 cells increased, whereas the number of CD8_s4, CD8_s5, CD8_s8, and CD8_s9 cells decreased (Fig. 4d). Using immunohistochemistry staining, we found that the number of CD8^+^ T cells increased after RFA treatment (Figure S2). The TCR analysis for CD8^+^ T cells showed that CD8_s2 had the highest number of clonotypes in both control group and RFA group, and highest percentage of cells were CD8_s1 occupied clonotype 1–5 (Fig. 5a and Table S2). There were three clusters (Cd8_s4, Cd8_s5, Cd8_s9) in RFA group that had several or none clonotypes owing to the lake of cells in these clusters. To gain further insight into the relationship among these nine CD8^+^ T clusters, we used Monocle 2 to analyze scRNA-seq data^26^. Such pseudo-time analysis is a measurement through biological processes based on transcriptional similarities^27^. Based on the expression signature, the starting point corresponded to the immune cells within CD8_s4 and CD8_s5, followed by CD8_s9, and then CD8_s2 and CD8_s3, and ended with CD8_s1, CD8_s6, CD8_s7, and CD8_s8 (Fig. 5b, c). Depending on the expression signatures, the four clusters at the late period of pseudo-time were exhausted T cells. CD8_s2 appeared to be a transitional state^26^ between naive and exhausted T cells based on trajectory and TCR analysis. These findings suggest that RFA treatment enhanced the antitumor immune response by promoting functional CD8 accumulation, and these effects were most evident in the antigen-specific CD8^+^ T cells.

### Changes in innate lymphoid cells and Mki67^high^ cells

Single-cell RNA-seq identified one cluster of innate lymphoid cells (ILC_s) (Fig. 4c), a cluster of cells lacking antigen-specific receptors and regulating immune response and tissue homeostasis^28^, and two cluster of Mki67^high^ cells (Mki67^hi^_s1 and Mki67^hi^_s2) (Fig. 4c), which expressed patterns of genes associated with T-cell proliferation^18^, including *Ezh2*, *Hmmr*, *Cenpa*, and *Mcm5* (Figure S1). Interestingly, cells of ILC_s and Mki67^hi^_s2 did not express *Cd4* and *Cd8b1* genes, whereas Mki67^hi^_s1 expressed both *Cd4* and *Cd8b1* genes (Fig. 4b). In ILC_s cluster, cells showed higher gene expression of *Il2ra*, *Icos*, *Gata3*, and *Rora* (Figure S1), suggesting ILC_s cluster was likely group 2 ILC_s^28,29^. The proportion of ILC_s cells decreased dramatically in RFA group compared with the control group (Fig. 4d). In Mki67^high^ clusters, Mki67^hi^_s1 contained a mixture of CD4^+^ T cells, CD8^+^ T cells, and Tregs based on their profound cell proliferation signature^18^, whereas Mki67^hi^_s2 cells lacked these cell signatures. The subpopulations of immune cells in Mki67^hi^_s1 were also altered after RFA therapy. The number of CD4^+^ T cells and CD8^+^ T cells in Mki67^hi^_s1 cluster were increased after RFA treatment (Fig. 5d, e), indicating RFA treatment may induce T-cell proliferation. Gene ontology and KEGG enrichment analysis revealed that Mki67^hi^_s1 displayed upregulation of pathways associated with ribosome and T-cell activation, whereas Mki67^hi^_s2 was associated with lysosome, T-cell activation and positive regulation of response to external stimulus pathways (Fig. 4e, f). These data suggest that RFA treatment could induce T-cell proliferation within the tumors.

### Changes in monocytes/macrophages

**Fig. 6:**
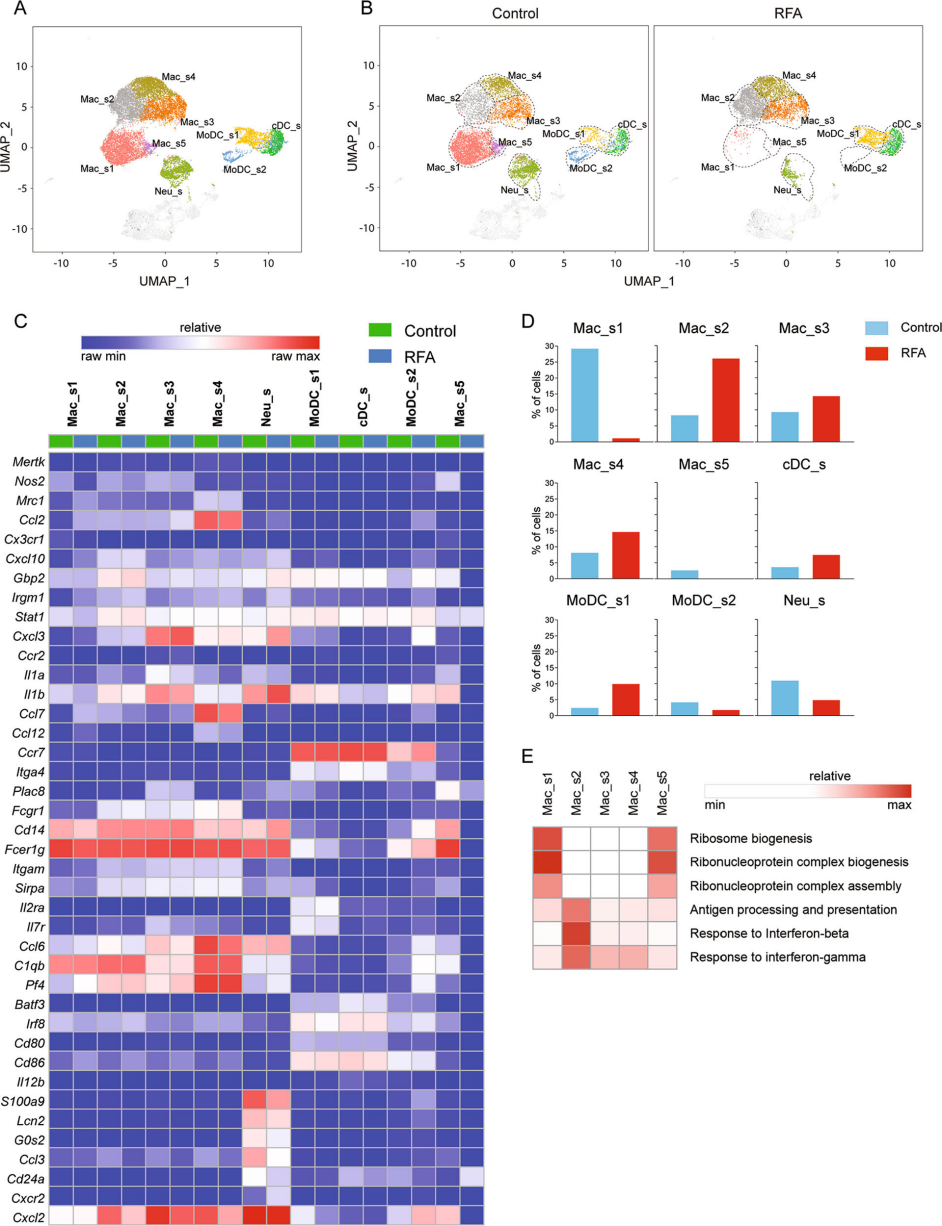
RFA induces intratumoral monocytes/macrophages, DCs, and neutrophils remodeling. **a** UMAP plots from merged data of these subpopulations. **b** UMAP plots with annotated clusters of these cells. **c** Heatmap displaying normalized expression of select markers. **d** Percentage of cells in each cluster by condition. **e** Heatmap of gene ontology enrichment analysis enrichment by clusters.

**Fig. 7:**
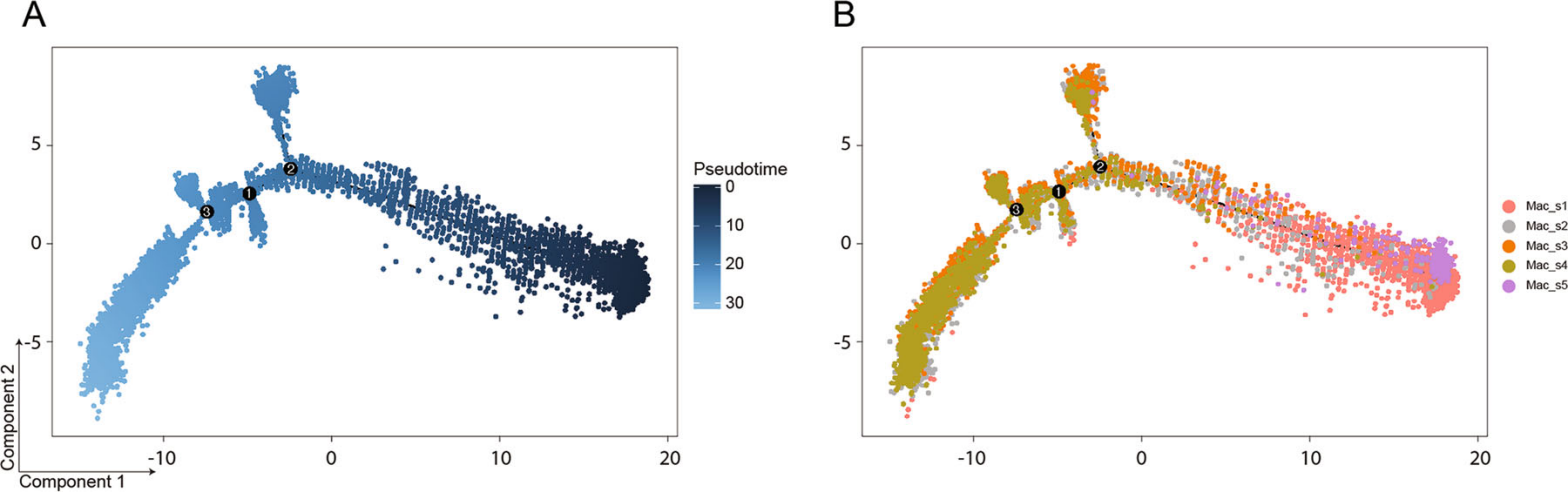
Biological processes of intratumoral monocytes/macrophages. **a** Trajectory plot with corresponding analysis of data by Monocle 2. **b** Seurat cluster of monocytes/macrophages clusters overlaid on Monocle 2 pseudo-time plot.

Intratumoral monocytes/macrophages represented the largest proportion of infiltrative immune cells in both control and RFA treatment group as revealed by scRNA-seq UMAP plots (Fig. 6a). Tumor-associated macrophages that expressed high levels of *Mrc1* (CD206) correlated with tumor immunosuppression, angiogenesis, metastasis, and relapse^30^. In contrast, monocytes/macrophages that expressed high levels of *Nos2* (iNOS) were associated with pro-inflammatory and antitumor functions^18^. In this study, scRNA-seq identified five major monocyte/macrophage subpopulations (Fig. 6b). Mac_s5 were characterized by the absence of classical macrophage maturation markers such as *Mertk*, but expressed the highest levels of *Ccr2*, *Ccr7*, *Itga4*, and *Plac8* (Fig. 6c), consistent with the monocyte phenotype^18^. The percentage of Mac_s5 decreased dramatically after RFA treatment (Fig. 6d). Mac_s1 and Mac_s4 cells were distinguished as having a distinctly anti-inflammatory gene-expression profile. Mac_s1 expressed genes encoding *Ccl2*, and most strikingly, *Cx3cr1* (Fig. 6c) and also expressed signatures concerning the ribosome biogenesis (Fig. 6e). The proportion of Mac_s1 also reduced obviously in RFA group (Fig. 6d). Mac_s4 cluster had higher expression of *Ccl2*, *Ccl7*, and *Ccl12* genes (Fig. 6c) and increased slightly in RFA group (Fig. 6d). In contrast, Mac_s2 and Mac_s3 were characterized as having distinctly antitumor function markers. Mac_s3 had higher expression of *Cxcl3*, *Ccr2*, *Il1a*, and *Il1b* genes (Fig. 6c). Mac_s2 expressed high levels of *Cxcl10*, *Gbp2*, *Irgm1*, and *Stat1*, which are related to the pathways of antigen processing and presentation, as well as the response to interferon-beta and interferon-gamma (Fig. 6e). In addition, the expression of *Nos2* was high in Mac_s2 and Mac_s3, whereas *Mrc1* was low. (Fig. 6c). The proportion of cells in Mac_s2 and Mac_s3 increased after RFA treatment (Fig. 6d), especially the Mac_s2 cluster. In addition, the number of CD206^+^ cells had barely changed and iNOS^+^ cells increased after RFA treatment in Immunohistochemical analysis (Figure S2). Further, we applied the Monocle 2 algorithm^26^ to order monocytes/macrophages in pseudo-time to indicate their developmental trajectories. All macrophage clusters formed into a relative process in pseudo-time that began with Mac_s5 cluster (monocytes), followed by Mac_s1 and then become macrophages in Mac_s2, Mac_s3, and Mac_s4 (Fig. 7a, b). Cells in Mac_s2, Mac_s3, and Mac_s4 overlapped with each other in trajectory plot, indicating that these three clusters had transcriptional similarities and might transform into each other based on the intratumoral stimulations. These data demonstrate that RFA treatment could stimulate macrophages to participate in forming an anti-TME.

### Changes in DCs and neutrophils

Single-cell RNA-seq revealed three distinct subsets of DCs, including one cluster of conventional DC (cDC_s) and two clusters of monocyte DC (MoDC_s1 and MoDC_s2) (Fig. 6a). cDC_s expressed high levels of Classical type 1 DC markers, including *Batf3* and *Irf8*. Both MoDC_s1 and MoDC_s2 expressed high levels of monocyte DC markers^31,32^, such as *Fcgr1*, *Cd14*, *Fcer1g*, *Itgam* (CD11b), and *Sirpa* (CD172). MoDC_s1 also had higher expression of *Il2ra* and *Il7r*, whereas MoDC_s2 expressed high levels of *Ccl6*, *C1qb*, and *Pf4*. All DC clusters displayed upregulation of the costimulatory molecules *Cd80*/*Cd86* and expression of *Il12b* (IL-12) that drive T-cell proliferation and differentiation (Fig. 6c). The proportions of cDC_s and MoDC_s1 increased after RFA treatment, whereas the proportion of MoDC_s2 decreased (Fig. 6d).

Lately, scRNA-seq also identified a neutrophil cluster (Neu_s) that expressed genes encoding *S100a9*, *Lcn2*, *G0s2*, *Ccl3*, and *Cd24a* (Fig. 6a, c). These cells also express high levels of *Cxcr2* and *Cxcl2* genes, which are associated with tumor progression and metastasis^33,34^. The percentage of Neu_s was reduced after RFA treatment (Fig. 6d). In summary, these findings demonstrate that RFA treatment increased the numbers of functional DCs, which stimulated T-cell proliferation and differentiation, and suppressed the function of tumor-associate neutrophils.

## Discussion

There is an increasing body of evidence that RFA could stimulate transient distant immune response. Combining this response with immunotherapy may stimulate enhanced antitumor activity^10,13,35,36^. However, most studies focused on the tumor of RFA side to understand the changes in the TME. Our study has demonstrated that RFA could result in a strong T-cell-mediated immune response in distant tumors^13^. To further assess the immune changes, we employed scRNA-seq and TCR profiles analysis approaches to profile immune cells infiltrating in distant non-RFA tumors after RFA treatment. In contrast to some previous studies^37^ that showed that incomplete ablation of a target tumor was associated with new metastases and poor survival and reduced efficacy of anti-PD-1 immunotherapy, our study did not detect the myeloid-derived suppressor cells in the distant non-RFA tumor after RFA treatment. Furthermore, our study presents a comprehensive view of the cellular dynamics induced by RFA in distant tumors.

Our study reveals several important new findings including the followings: (1) RFA was capable of enhancing antitumor immunity in distant non-RFA tumors. (2) The immunosuppressive components in the TME including Tregs, tumor-associated macrophages and tumor-associated neutrophils were suppressed after RFA treatment. (3) RFA treatment induced the differentiation and maturation of naive immune cells and increased the proliferative capability of T cells in distant non-RFA tumors. (4) More functional DCs were detected in the tumors after RFA treatment. (5) CD4^+^ and CD8^+^ T cells were enriched in the tumor tissues of non-RFA tumor after RFA treatment, however, the majority of these cells were exhausted phenotypes.

We also found that RFA increased the infiltration of CD8^+^PD-1^+^ T cells in distant non-RFA tumor on day 3 after RFA treatment, whereas at the same time upregulated the expression of PD-1 and LAG3, the critical immune checkpoint regulators in the T cells. The upregulated expression of PD-1 and LAG3 may lead to immune suppressive effect in the TME, causing its failure to shrink the tumor. In addition, the percentage of CD8^+^PD-1^+^ T cells decreased quickly on days 5 and 8. Hence, although we detected the decreased numbers of Tregs, tumor-associated macrophages and tumor-associated neutrophils, and increased numbers of functional DCs, CD4^+^, and CD8^+^ T cells on day 3 after RFA treatment, the transient immune responses lacked the ability to suppress tumor growth.

Some previous studies^10,36,38^ revealed that combination of RFA with immune checkpoint inhibitor could enhance antitumor immunity on the RFA-treated tumors, but few studies^13^ showed RFA in combination with anti-PD-1 antibody could result in a synergistic antitumor effect in distant non-RFA-treated tumors. In this study, we observed the upregulation of PD-1 (*Pdcd1*) in CD4^+^ and CD8^+^ T cells, as well as upregulated LAG3 in T cells after RFA treatment, indicating that T-cell exhaustion was prevalent at the distant tumors.

In conclusion, our study provides new insight into the transcriptional, molecular, and functional changes in the non-RFA distant tumors induced by RFA treatment, and discovered that T-cell exhaustion after RFA was likely the mechanism for the failure of the RFA to induce effective tumor shrinkage on the distant non-RFA tumors. However, our data suggest that immune checkpoint inhibitors, when combined with RFA, may have the potential to overcome the T-cell exhaustion and enhance the activities of the transiently increased infiltrating cytotoxic T-cell in the distant tumors to exert effective tumor shrinkage.

## Supplementary Information

Supplementary Figures

Supplementary Figure S1

Supplementary Figure S2

Supplementary Table S1

Supplementary Table S2

## Data Availability

Single-cell RNA-seq data that support the findings of this study have been deposited in GEO with the accession code PRJNA611464 and Main R code for the analysis are available at GitHub.
