# Supplementary material for: High-dimensional single-cell analysis delineates radiofrequency ablation induced immune microenvironmental remodeling in pancreatic cancer
Source: Cell Death Dis. 2020 Jul 27;11(7):589. doi: 10.1038/s41419-020-02787-1 (PMC7385122; doi:10.1038/s41419-020-02787-1)
Supplement: Supplementary file 1 — Supplementary Figures [file 41419_2020_2787_MOESM1_ESM.docx]

**Figure S1.** Heatmap displaying normalized expression of select markers in lymphocyte subpopulations.

**Figure S2.** Immunohistochemistry staining of CD4, CD8, FOXP3, CD206 and iNOS in distant non-RFA tumors. (A) Immunohistochemistry staining with anti-CD4, anti-CD8, anti-FOXP3, anti-CD206 and anti-iNOS antibody in distant non-RFA tumors (n = 3) at low (100×) and high magnification (400×). Scale bar = 50 μm (red line at the bottom left). (B) Results of immunohistochemical staining.
