# Supplementary figures and images for: High-dimensional single-cell analysis delineates radiofrequency ablation induced immune microenvironmental remodeling in pancreatic cancer
Source: Cell Death Dis. 2020 Jul 27;11(7):589. doi: 10.1038/s41419-020-02787-1 (PMC7385122; doi:10.1038/s41419-020-02787-1)

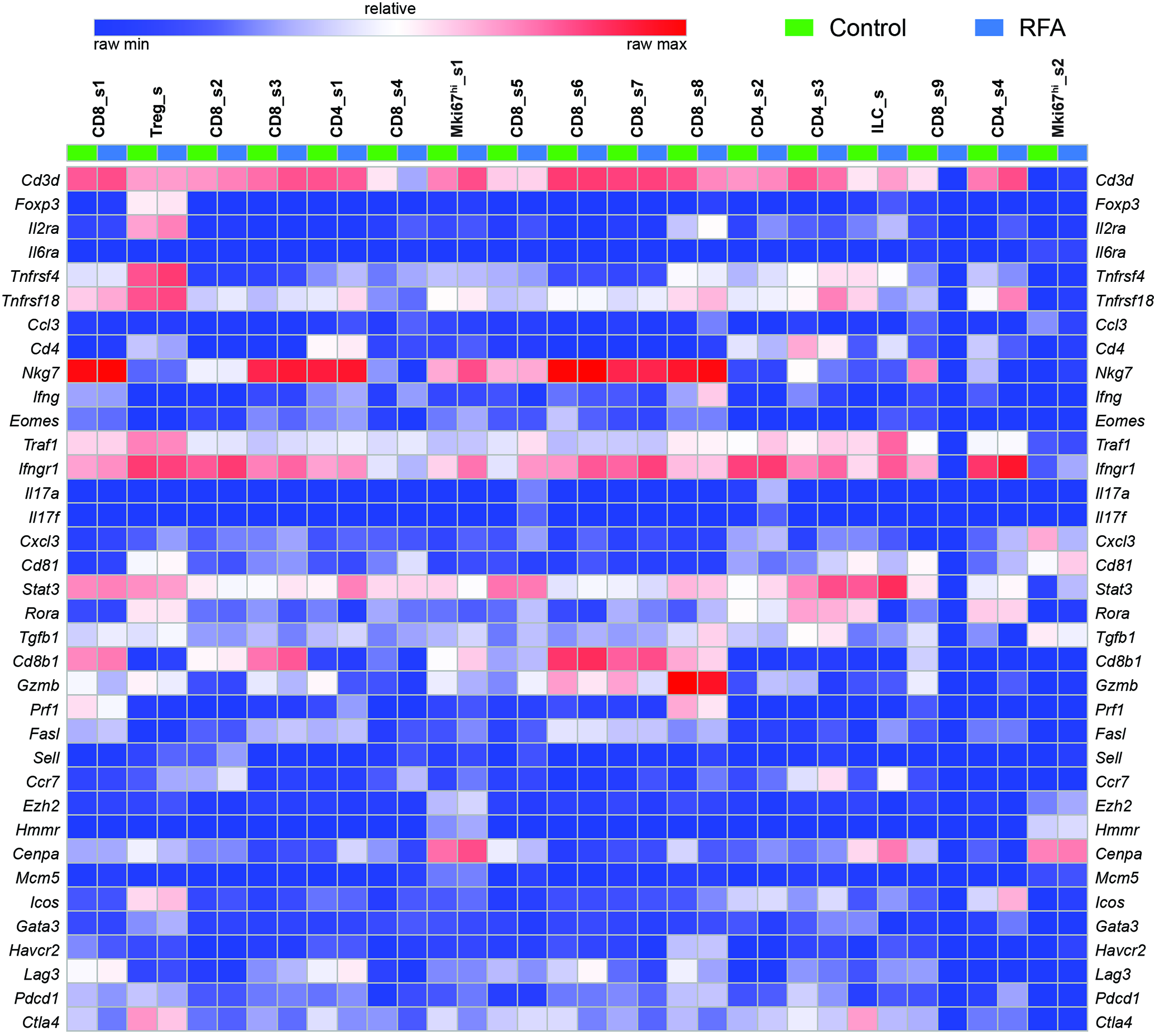

Supplement: Supplementary file 2 — Supplementary Figure S1 [file 41419_2020_2787_MOESM2_ESM.tif]

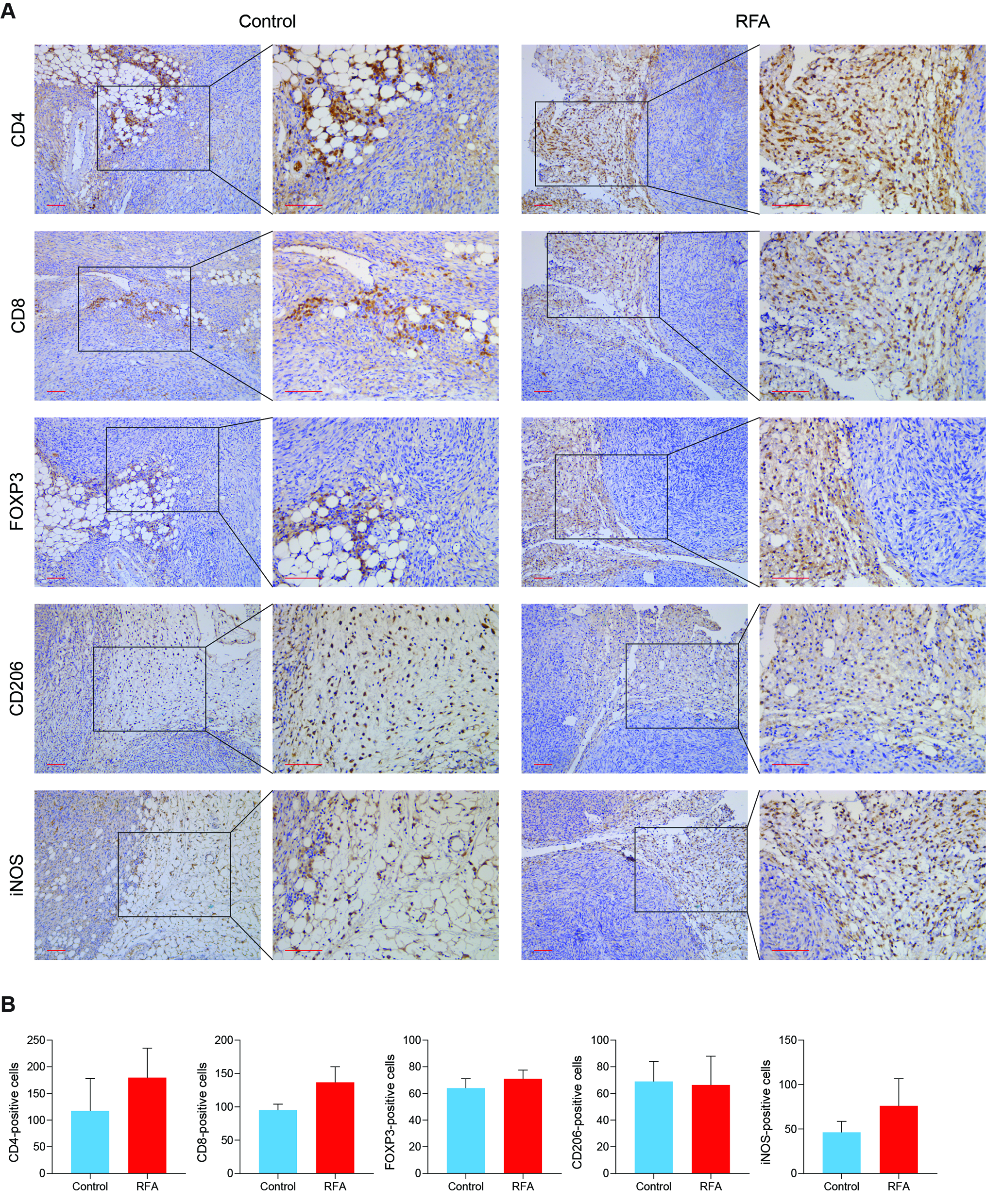

Supplement: Supplementary file 3 — Supplementary Figure S2 [file 41419_2020_2787_MOESM3_ESM.tif]
