# Supplementary material for: High-dimensional single-cell analysis delineates radiofrequency ablation induced immune microenvironmental remodeling in pancreatic cancer
Source: Cell Death Dis. 2020 Jul 27;11(7):589. doi: 10.1038/s41419-020-02787-1 (PMC7385122; doi:10.1038/s41419-020-02787-1)
Supplement: Supplementary file 4 — Supplementary Table S1 [file 41419_2020_2787_MOESM4_ESM.docx]

Table S1 Details of clonotypes in each cluster by condition

|  | control group | | | RFA group | | |
| --- | --- | --- | --- | --- | --- | --- |
| cluster | lymphoid cell | clonotype | frequency | lymphoid cell | clonotype | frequency |
| Treg_s | 257 | 91 | 119 | 160 | 71 | 79 |
| CD4_s1 | 133 | 27 | 101 | 140 | 34 | 110 |
| CD4_s2 | 72 | 32 | 37 | 70 | 27 | 27 |
| CD4_s3 | 73 | 30 | 47 | 37 | 21 | 26 |
| CD4_s4 | 44 | 20 | 32 | 3 | 3 | 3 |
| CD8_s1 | 216 | 37 | 182 | 385 | 92 | 284 |
| CD8_s2 | 190 | 82 | 99 | 167 | 106 | 109 |
| CD8_s3 | 184 | 38 | 139 | 139 | 41 | 111 |
| CD8_s4 | 236 | 58 | 62 | 14 | 4 | 4 |
| CD8_s5 | 206 | 41 | 70 | 14 | 1 | 1 |
| CD8_s6 | 43 | 14 | 37 | 165 | 57 | 153 |
| CD8_s7 | 81 | 33 | 60 | 102 | 65 | 90 |
| CD8_s8 | 127 | 30 | 107 | 24 | 13 | 14 |
| CD8_s9 | 55 | 26 | 33 | 0 | 0 | 0 |
| Mki67^hi^_s1 | 130 | 68 | 93 | 97 | 66 | 84 |
| Mki67^hi^_s2 | 8 | 0 | 0 | 32 | 2 | 2 |
| ILC_s | 85 | 14 | 18 | 2 | 2 | 2 |
