# Supplementary material for: High-dimensional single-cell analysis delineates radiofrequency ablation induced immune microenvironmental remodeling in pancreatic cancer
Source: Cell Death Dis. 2020 Jul 27;11(7):589. doi: 10.1038/s41419-020-02787-1 (PMC7385122; doi:10.1038/s41419-020-02787-1)
Supplement: Supplementary file 5 — Supplementary Table S2 [file 41419_2020_2787_MOESM5_ESM.docx]

Table S2 Percentage of cells in clonotype 1-5 in each cluster by condition

|  | control group | | RFA group | |
| --- | --- | --- | --- | --- |
| cluster | clonotype | percentage of cells in  clonotype 1-5 | clonotype | percentage of cells in  clonotype 1-5 |
| CD4_s1 | 27 | 72.28% | 34 | 65.45% |
| CD4_s2 | 32 | 27.03% | 27 | 18.52% |
| CD4_s3 | 30 | 46.81% | 21 | 38.46% |
| CD4_s4 | 20 | 53.13 % | 3 | -* |
| CD8_s1 | 37 | 76.37% | 92 | 53.17% |
| CD8_s2 | 82 | 18.18% | 106 | 7.34% |
| CD8_s3 | 38 | 64.03% | 41 | 49.55% |
| CD8_s4 | 58 | 14.52% | 4 | -* |
| CD8_s5 | 41 | 40.00% | 1 | -* |
| CD8_s6 | 14 | 70.27% | 57 | 41.83% |
| CD8_s7 | 33 | 43.33% | 65 | 28.89% |
| CD8_s8 | 30 | 66.36% | 13 | 42.86% |
| CD8_s9 | 26 | 36.36% | 0 | -* |

* The number of clonotype in these cluster less than 5. The percentage of cells in clonotype 1-5 is meaningless compared to other clusters.
